# Supplementary material for: Potent activity of polymyxin B is associated with long-lived super-stoichiometric accumulation mediated by weak-affinity binding to lipid A
Source: Nat Commun. 2024 Jun 3;15:4733. doi: 10.1038/s41467-024-49200-5 (PMC11148078; doi:10.1038/s41467-024-49200-5)
Supplement: Supplementary file 1 — Supplementary Information [file 41467_2024_49200_MOESM1_ESM.pdf]

Supplementary Information

Potent activity of polymyxin B is associated with long-lived super-stoichiometric accumulation mediated by weak-affinity binding to lipid A

Kerry R. Buchholz[1]*, Mike Reichelt[2], Matthew C. Johnson[3], Sarah J. Robinson[4], Peter A. Smith[1,5], Steven T. Rutherford[1]*, John G. Quinn[6]*

[1] Department of Infectious Diseases, Genentech, Inc., South San Francisco, CA, USA
[2] Department of Pathology, Genentech, Inc., South San Francisco, CA, USA
[3] Department of Structural Biology, Genentech, Inc., South San Francisco, CA, USA
[4] Department of Discovery Chemistry, Genentech, Inc., South San Francisco, CA, USA
[5] Current address: Revagenix, Inc., San Mateo, CA, USA
[6] Department of Biochemical and Cellular Pharmacology, Genentech, Inc., South San Francisco, CA, USA
*Address correspondence to buchholz.kerry@gene.com, quinn.john@gene.com, and rutherford.steven@gene.com

Contents **Pages**

- SI Fig. S1-S11 2-12
- SI Section 1 (including SI Fig. S12-S16) 13-22
- SI Section 2 23-26
- SI Methods 27-29
- SI Tables S1-S5 30-34
- SI References 35-36

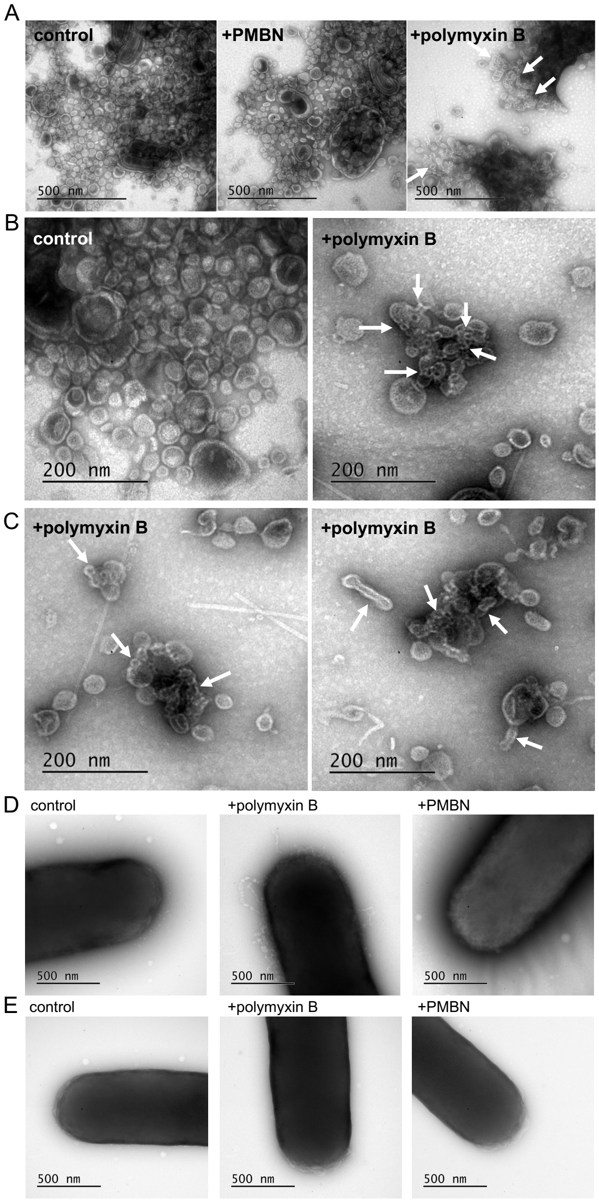

SI Fig. S1. Transmission electron microscopy (TEM) analysis of OMVs. (**A**) Representative TEM images of wt-OMVs exposed to buffer (left), PMBN (middle), or polymyxin B (right) for 40 minutes. Scale bars = 500 nm. (**B**) Uncropped TEM images from **Fig. 1B** of wt-OMVs exposed to buffer (left) or polymyxin B (right). Scale bars = 200 nm (**C**) Additional representative images of wt-OMVs exposed for polymyxin B for 40 minutes. Scale bars = 200 nm. White arrows (A-C) point to sites of microvesiculation or tubules. (D and E). TEM are representative of n=3 biological replicates for untreated and polymyxin B-treated OMVs and n=2 biological replicates for PMBN-treated OMVs. 10-20 images were taken for each sample. Representative TEM images of (**D**) wt-*E. coli* or (**E**) resistant *E. coli* (pBAD-*mcr*1) cells treated with buffer (left), polymyxin B (middle), or PMBN (right) for 40 minutes, (D-E) Scale bars = 500 nm. n=3 biological replicates were performed for polymyxin B-treated wt-*E. coli* and n=2 biological replicates were performed for PMBN-treatment and polymyxin B-treated pBAD-*mcr*1. 10-20 images were taken for each sample.

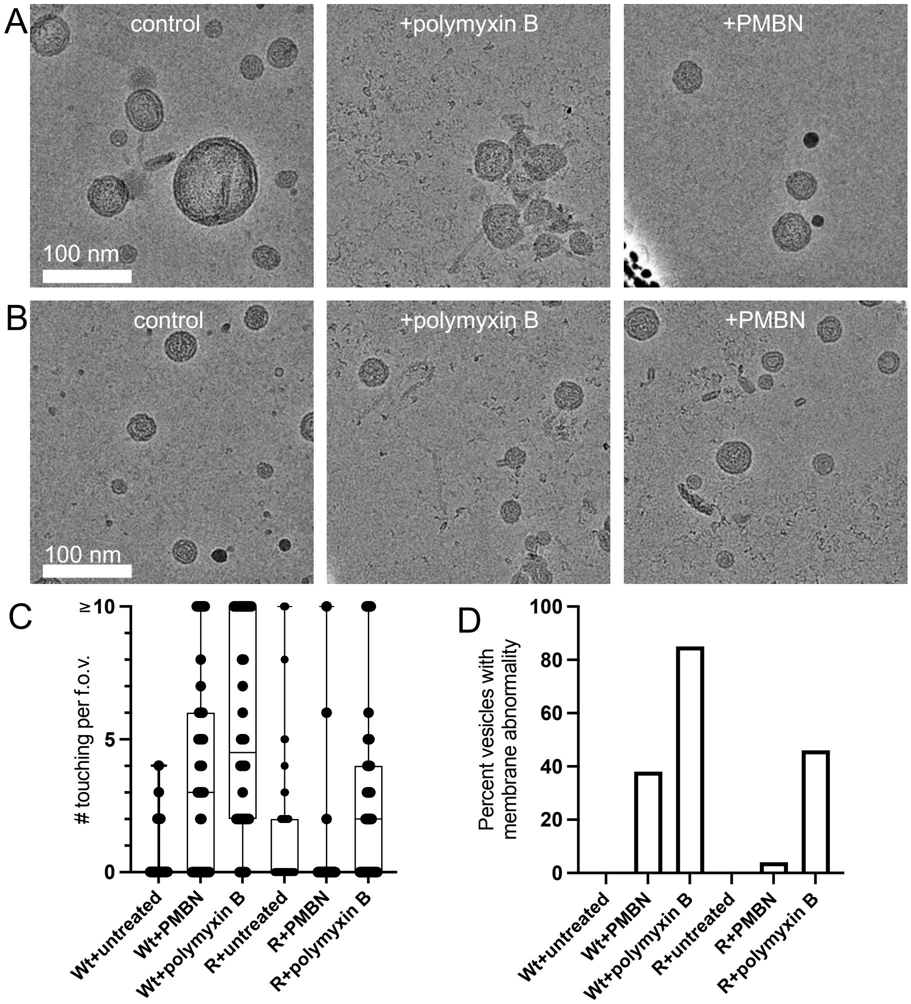

SI Fig. S2. Cryo-electron microscopy of OMVs. Representative images of (**A**) wt-OMVs or (**B**) resistant-OMVs (PmrA^{G53E}) exposed to buffer (control, left), polymyxin B (middle), or PMBN (right) for 20 minutes. Representative of more than 47 images. Polymyxin B and PMBN were added to a final ratio with LPS of ~0.5:1. Scale bars = 100 nm. Images have been low-pass filtered and down-sampled to enhance contrast. (**C**) Quantification of cryo-electron microscopy of OMVs. 50 images each, 47 for Wt+PMBN, were blinded and scored for (**C**) number of vesicles touching (0-10+), whiskers are min to max, all data points shown, line indicates median. (**D**) percent of images analyzed where any vesicle in the field of view had abnormal membrane appearance. Images used were selected randomly but curated such that those selected all contained at least one vesicle. Areas with large amount of vesicle clumping (occurring with polymyxin B treatment) could not be imaged. PMBN – polymyxin B nonapeptide, Wt - OMVs from wild-type *E. coli*, R - OMVs from polymyxin-resistant *E. coli*. Full data and image sets are in the Source Data file.

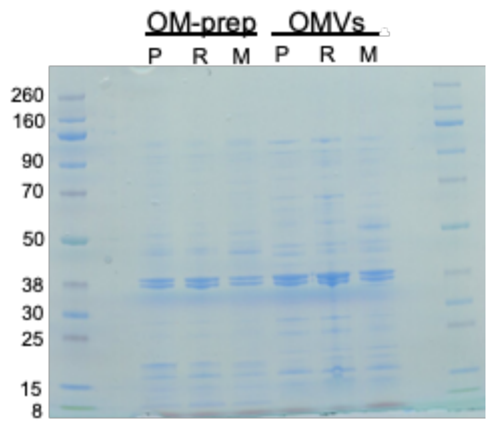

SI Fig. S3. SDS-PAGE analysis of *E. coli* outer membrane preparations (OM-prep) and OMVs stained with Coomassie Blue. P - parental wild-type *E. coli* strain (unmodified lipid A), R - polymyxin-resistant *E. coli* PmrA^{G53E}, M - polymyxin-resistant *E. coli* with pBAD-*mcr1*.

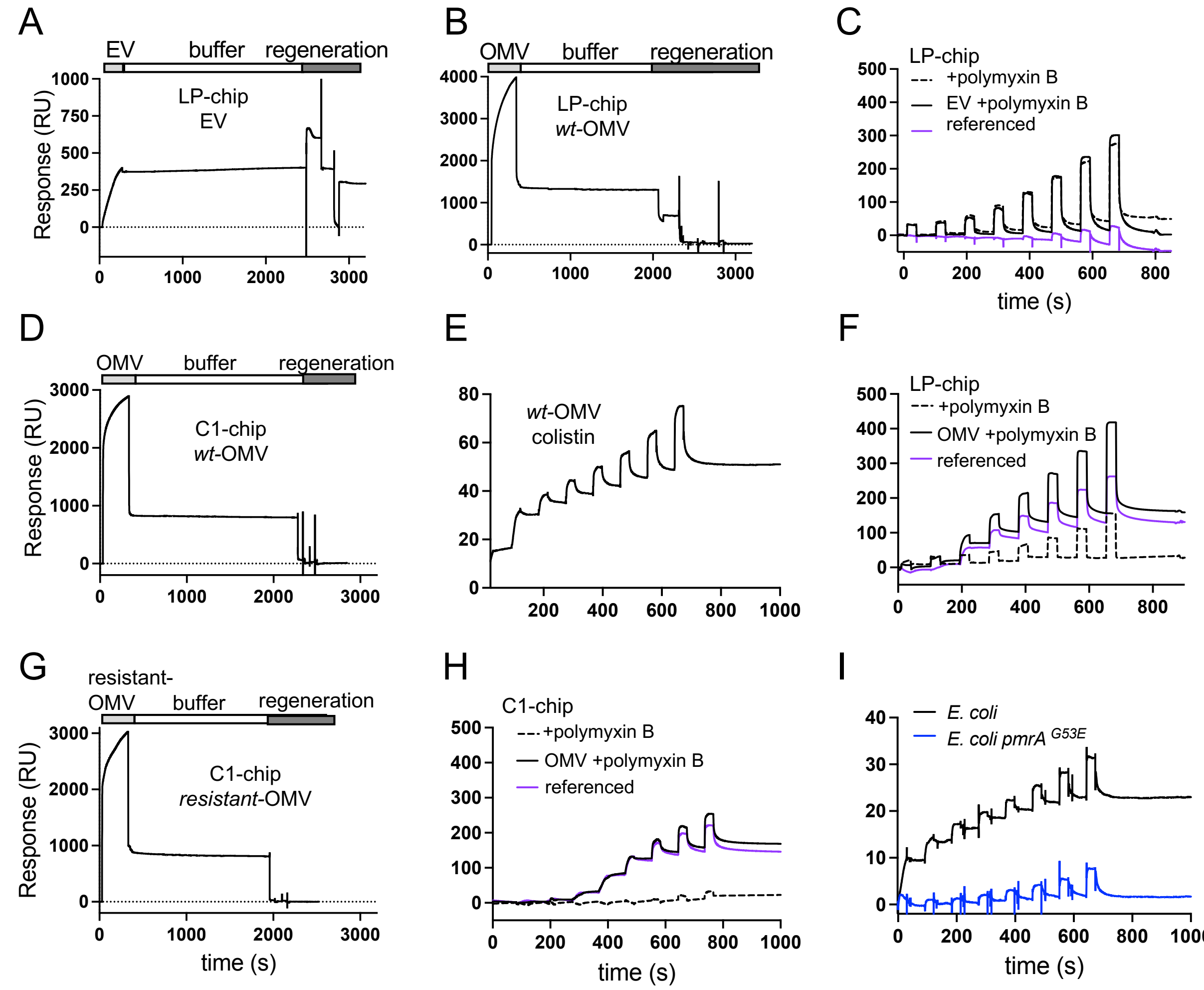

SI Fig. S4. SPR sensorgrams for binding of polymyxin B to mammalian vesicles, bacterial OMVs, and whole cells using lipophilic LP chips and via amine-coupled polymyxin B to the surface of C1 chips. (**A**) Representative SPR sensorgram of mammalian extracellular vesicles loading onto LP chips and regeneration. (**B**) Representative SPR sensorgram of wt-OMVs loading onto LP chips and regeneration. (**C**) Representative SPR sensorgrams of polymyxin (312.5 nM – 40 µM) over mammalian vesicles on LP chip. 'Referenced' indicates subtraction of binding on blank chip surface from binding to EV-loaded surface. 1272 RU loaded. (**D**) Representative SPR sensorgram of wt-OMVs loading onto C1 chip via amine-coupled polymyxin B followed by regeneration. (**E**) SPR binding curves showing a stepwise increase in binding response upon exposure of affinity-captured wt-OMVs to eight serial-doubling concentrations of colistin from 39 nM – 5 µM. 362 RU loaded. (**F**) Representative SPR sensorgrams of polymyxin B (312.5 nM – 40 µM) over wt-OMVs on LP chip. 1049 RU loaded. (**G**) Representative SPR sensorgram of resistant-OMVs isolated from *E. coli* PmrA^{G53E} cells loading onto amine-coupled polymyxin B-C1 chip and regeneration. **(H)** Representative SPR sensorgrams of polymyxin (39 nM – 5 µM) over OMVs loading onto amine-coupled polymyxin B-C1 chip. 'Referenced' indicates subtraction of binding on blank chip surface from binding to OMV-loaded surface. 1449 RU loaded. (**I**) Example of SPR sensorgram of polymyxin B (39 nM – 5 µM) binding to whole cells of wild-type *E. coli* (137 RU loaded) or polymyxin B-resistant *E. coli* PmrA^{G53E} (55 RU loaded). Note that lower loading does not affect overall conclusions when compared to wild-type bacteria but does affect the magnitude of the RUs. All displayed curves are representative of n≥3 independent replicates.

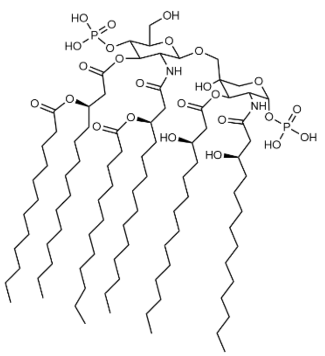

Lipid A

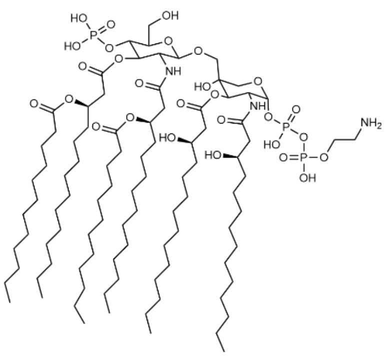

Singly phosphoethanolamine (pEtN) modified Lipid A

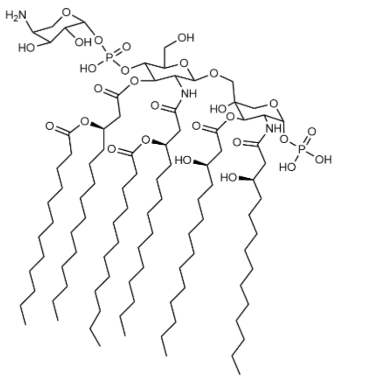

Singly 4-amino-4-deoxy-L-arabinose (L-Ara4N) modified Lipid A (only one shown)

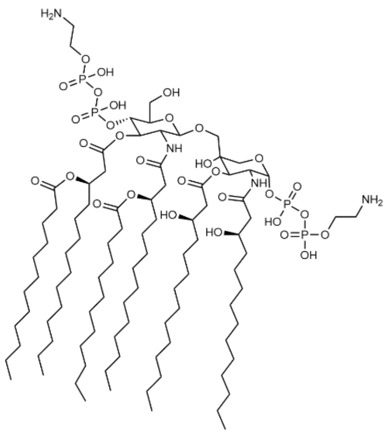

Doubly pEtN modified Lipid A

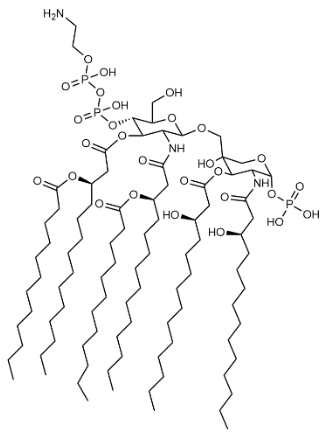

Singly pEtN modified Lipid A

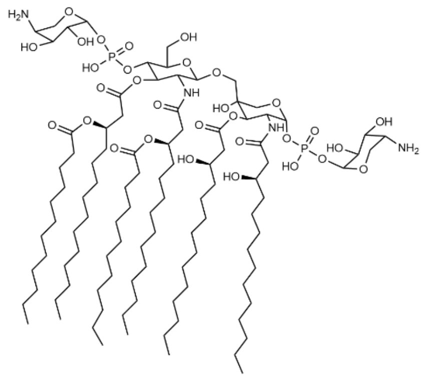

Doubly L-Ara4N modified Lipid A

SI Fig. S5. Chemical structures of lipid A and lipid A with polymyxin-resistant modifications.

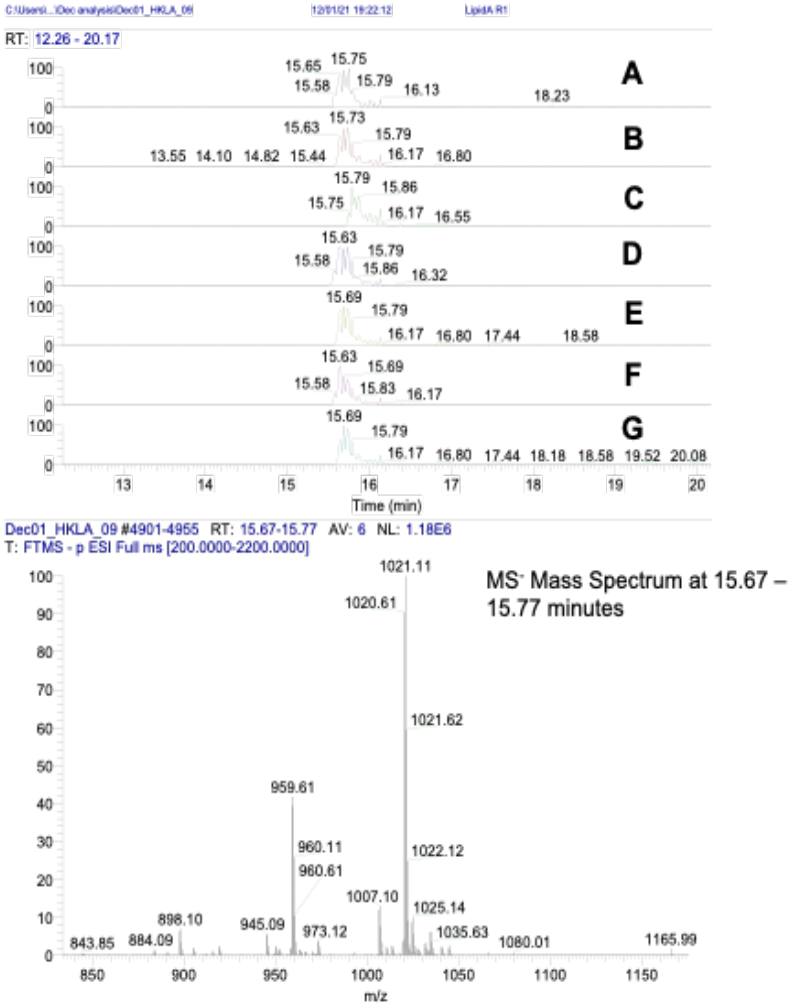

SI Fig. S6. Lipid A R1 MS^- extracted ion chromatograms (EIC) of lipid A and phosphoethanolamine (pEtN) modifications thereof as annotated (top). pEtN EIC mass ranges, peak intensity, and identification are as follows: (**A**) EIC *m/z* 884.07 – 884.10, 3.16E4, unmodified lipid A; (**B**) EIC *m/z* 898.08 – 898.12, 1.40E5, unmodified lipid A + C_2H_4; (**C**) EIC *m/z* 919.60 – 919.64, 2.73E5, unmodified lipid A + C_5H_{10}; (**D**) EIC *m/z* 945.07 – 949.11, 1.14E5, singly pEtN-modified lipid A; (**E**) EIC *m/z* 959.58 – 959.62, 8.40E5, singly pEtN-modified lipid A + C_2H_4; (**F**) EIC *m/z* 1007.07 – 1007.11, 3.65E5, doubly pEtN-modified lipid A; and (**G**) EIC *m/z* 1021.09 – 1021.13, 2.08E6, doubly pEtN-modified lipid A + C_2H_4.The same data processing was applied to lipid A R1 MS^- LCMS data for pEtN plus 4-amino-4-deoxy-L-arabinose (L-Ara4N) doubly modified and L-Ara4N-modified lipid A. L-Ara4N mass ranges, peak intensity, and identification are as follows (chromatograms not shown): (1) EIC m/z 949.59-949.63, 7.82E5, singly L-Ara4N-modified lipid A; (2) EIC m/z 963.61 - 963.65, 5.47E5, singly L-Ara4N-modified lipid A + C_2H_4; (3) EIC m/z 1012.11 - 1012.15, 5.12E5, doubly pEtN plus L-Ara4N-modified lipid A; (4) EIC m/z 1026.12 - 1026.16, 2.69E6, doubly pEtN plus L-Ara4N-modified lipid A + C_2H_4; (5) EIC m/z 1016.13 - 1016.17, 1.26E5, doubly L-Ara4N-modified lipid A; and (6) EIC m/z 1030.15 - 1030.19, 0, doubly L-Ara4N-modified lipid A + C_2H_4. Lipid A R1 MS^- mass spectrum exhibiting relative intensities of doubly charged lipid A and modified ions (bottom). High-resolution, accurate mass data of pEtN and L-Ara4N modified lipid A analogs were acquired and analyzed (see SI Fig. S5 for chemical structures and Source Data for full spectra).

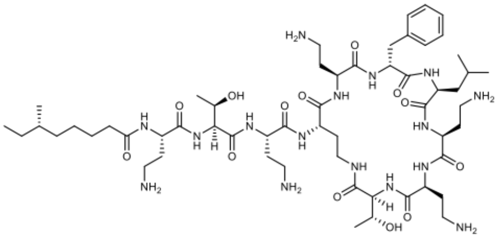

Polymyxin B

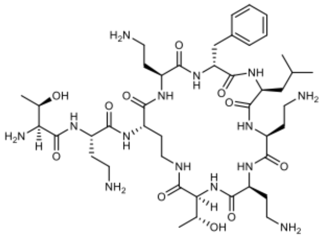

PMB nonapeptide

SI Fig. S7. Chemical structures of antibiotic polymyxin B (left) and the polymyxin B derivative, polymyxin B nonapeptide (PMBN), that lacks antibacterial activity (right).

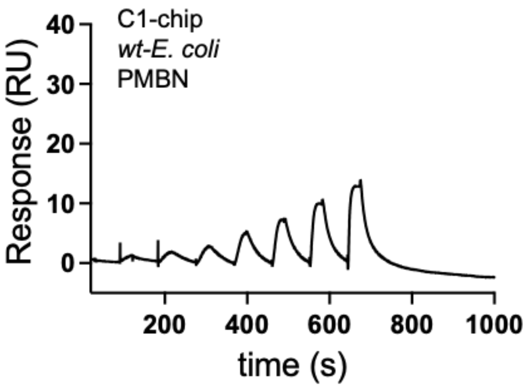

SI Fig. S8. SPR sensorgram of PMBN binding to bacterial cells. Representative SPR sensorgram of PMBN binding with wild-type *E. coli* cells (399 RU loaded) performed as described in **Fig. 2**. Representative of n=4 independent replicates.

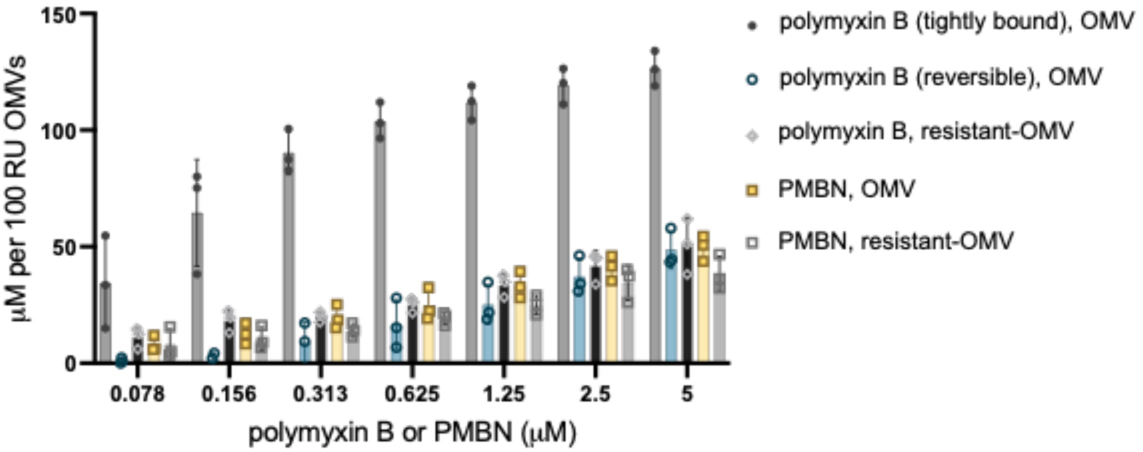

SI Fig. 9. Concentrations of polymyxin B or PMBN bound expressed per 100 RU of wt-OMVs and resistant-OMVs to normalize for loading differences, calculated from double-referenced sensorgrams and assuming 100 RU = 1 mg/ml. Mean and standard deviation for n=3 replicates are shown for each condition.

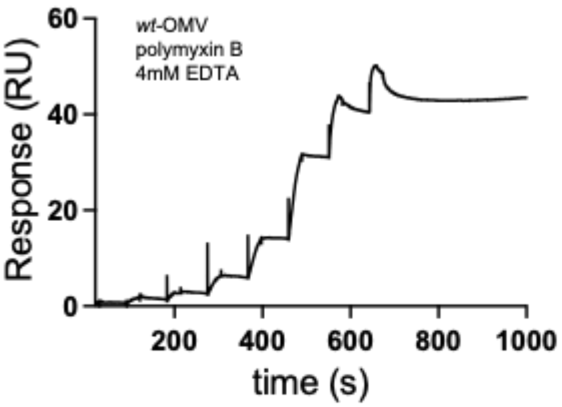

SI Fig. 10. Representative SPR sensorgram of polymyxin B binding to wt-OMVs in the presence of the metal chelator EDTA (4 mM). Each serial injection profile was recorded as outlined in Figure 1 with a maximum concentration of 625 nM. 401 RU OMV was loaded. Representative of n=3 independent replicates.

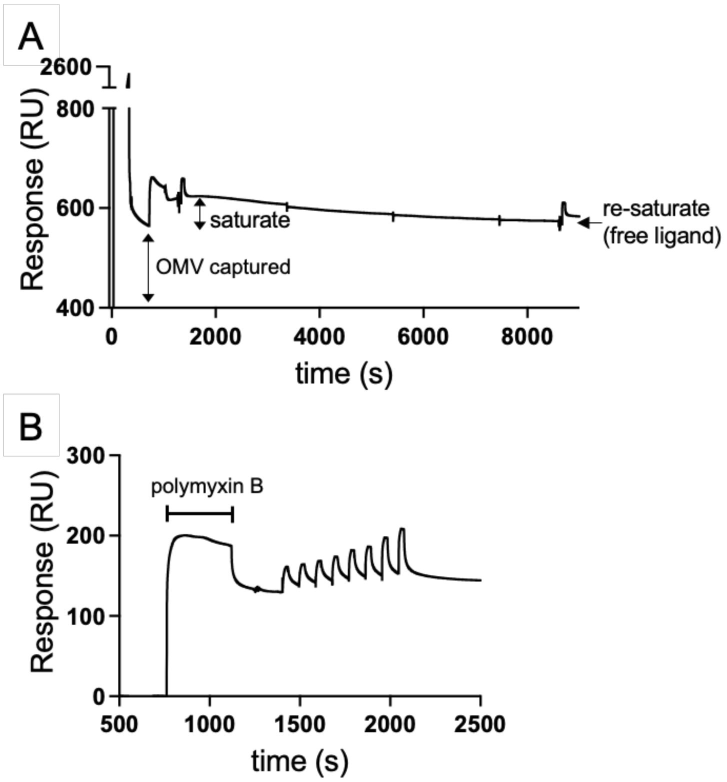

SI Fig. S11. Representative SPR sensorgrams used for approximation of polymyxin B interaction constants. (**A**) Chaser analysis. Saturation of wt-OMVs with polymyxin B is followed by re-saturation after a delay. (**B**) Affinity analysis. Estimation where saturation of wt-OMVs with polymyxin B is followed with serial injections of increasing concentrations of polymyxin B (39 nM - 5 μM) are performed. Representative of n≥8 independent replicates.

Section 1 - Mechanistic studies of polymyxins

Section 1A - Modeling

The interaction models introduced here (*Eqn (S1-S3)*) are annotated in terms of binding of polymyxin B (PMB) to LPS but also apply to any other affinity binding pair. Molar concentrations of bound PMB are converted to SPR response as R = G.MW.**[PMB]**, where MW is the molecular weight of PMB, and G is a mass-to-response conversion factor. A full quantitative interpretation of SPR sensitivity has been reported[1]. In a general case, a protein concentration of 0.01 (g/L) produces a response of 1 response unit (RU) yielding the proportionality constant G=100 (L/g). Here, we assume protein is distributed uniformly within a 100 nM thick hydrogel, dissolved in buffer at near physiological salt concentrations in the absence of high concentrations of high refractive index substances, such as glycerol and DMSO. This calibration may be adjusted for variability in the refractive index increment when working with buffers or other classes of molecules such as nucleic acids, polysaccharides, or drug-like molecules. In addition, binding interactions confined to planar sensing surfaces (i.e., no hydrogel matrix) results in a 1.3-fold increased response due to minimal decay in SPR sensitivity when close to the sensing surface (e.g., < 10 nm). Change in protein accumulation over time produces responses that are indicated here by a time subscript (e.g., $\mathbf{R_{(t)}}$). A simple 1:1 model for binding of PMB to LPS to form an affinity complex PMBL is given by the pseudo-first-order model defined by *Eqn (S1)*.

$$d\mathbf{R}/dt = (k_{on}.\mathbf{[PMB]_i}.(\mathbf{R_{max}}\text{-}\mathbf{R}_{(t)})\ \text{-}k_{off}.\mathbf{R}_{(t)}) \qquad \textit{Eqn (S1)}$$

Where **R** is the SPR response for accumulation of affinity complex **PMBL**. $\mathbf{[PMB]_i}$ is the injected concentration of PMB and $\mathbf{R_{max}}$ is the saturation response assuming full target occupancy. This model assumes that mass transport of PMB within the flow cell, which governs the rate of exchange of PMB between the bulk liquid and the sensing surface, is non-limiting. However, this assumption does not hold in many cases requiring addition of a mass transport term which results in the 1:1 two-compartment model in *Eqn (S2)*.

$$d\mathbf{R}/dt = (k_{on}.\mathbf{[PMB]}.(\mathbf{R_{max}}\text{-}\mathbf{R}_{(t)})\ \text{-}k_{off}.\mathbf{R}_{(t)}) + k_t.(\mathbf{[PMB]_{i(t)}} - \mathbf{[PMB]}) \qquad \textit{Eqn (S2)}$$

This model allows estimation of kinetic parameters despite the influence of mass transport limitation, where k_t (units, RU/Ms) = k_t'.100.MW_{PMB}.R_{max}. However, when mass transport constant k_t is entirely dominant then any observable curvature will be due to mass transport limitation and sensitivity to kinetic binding constants is lost entirely. In this extreme case *Eqn (S2)* simplifies to *Eqn (S3)*, which we refer to as a diffusion boundary model[2].

$$d\mathbf{R}/dt = k_t.\mathbf{[PMB]_{i(t)}} - k_t.K_D/(\mathbf{R_{max}}/\mathbf{R_t} - 1) \qquad \textit{Eqn (S3)}$$

Where the dissociation affinity constant $K_D = k_{off}/k_{on}$

Importantly, the kinetics of binding does not appear in *Eqn (S3)* because the observable kinetic curvature is associated with formation and decay of the mass transport boundary layer and not binding kinetics. In general, *Eqn (2)* can be used even when mass transport limitation is negligible and, in that case, k_t will tend towards infinity and, therefore, will not influence estimation of the kinetic binding constants. However, fitting of *Eqn (2)* in cases where binding is completely dominated by mass transport limitation is problematic because the goodness-of-fit, kinetic rate constants, and associated standard error can sometimes appear reasonable but in reality, the returned rate constants may remain entirely driven by boundary layer kinetics and not related to binding rate constants. Conditions requiring application of *Eqn (3)* can be defined using the Damköhler number (Da) as full transport limitation can be assumed when the binding flux Lr =

k_{on}*($\mathbf{R_{max}}$-$\mathbf{R_{(t)}}$) >> k_t, which is equivalent to $Da = k_t / L_r >> 1$. Therefore, a high k_{on} combined with a high surface binding capacity ($\mathbf{R_{max}}$-$\mathbf{R_{(t)}}$) will likely result in Da <<1. Increasing k_t would overcome this limitation and restore sensitivity to kinetic binding but this is usually not possible in practice because k_t cannot be increased by orders of magnitude since it is a function of flow cell geometry (which is fixed), analyte diffusion (which is fixed for a given experimental condition) and is weakly dependent on flow rate[3]. However, overcoming complete mass transport limitation is possible by lowering L_r by limiting the concentration of target at the sensing surface.

Polymyxin B (PMB) binding to whole *E. coli* cells, OMVs, and LPS forms transient complexes with available LPS in a fully transport limited regime and a fraction of these transient complexes (**R1**) transform into long-lived complexes (**R2**). Here, we neglect *Eqn (S3)* and crudely approximated the observed binding as an isomerization process, using a simple two-state model to provide a first approximation.

$$d\mathbf{R1}/dt = k_t.\mathbf{[PMBi]}_{(t)} - k_t.K_{D1}/(\mathbf{R_{max1}}/\mathbf{R1_t} - 1) - (k_{on2}.\mathbf{R1_{(t)}} - k_{off2}.\mathbf{R2_{(t)}}) \quad \textit{Eqn (S4)}$$

$$d\mathbf{R2}/dt = (k_{on2}.\mathbf{R1_{(t)}} - k_{off2}.\mathbf{R2_{(t)}}) \quad \textit{Eqn (S5)}$$

$$\text{Total Response } \mathbf{R_{(t)}} = \mathbf{R1_{(t)}} + \mathbf{R2_{(t)}} \quad \textit{Eqn (S6)}$$

This two-state approximation does not correctly account for the finite capacity of LPS-containing surfaces to retain PMB for extended periods. Changes in PMB occupancy can be obtained indirectly from SPR binding using chaser SPR analysis (see **Section 1D - Multipoint Chaser Binding**). Briefly, repeated PMBN injections over a sensing surface that has been pre-saturated with PMB are exploited to report loss in PMB occupancy over an extended time period. Progressive dissociation of PMB causes a corresponding increase in PMBN binding since PMBN binding is proportional to free LPS and therefore varies inversely with PMB dissociation. These time course measurements allow a PMB dissociation curve to be plotted and fit without interference from baseline drift. A simple 1:1 binding interaction produces a single exponential decay curve while biphasic decay curves are observed when there are two concurrent dissociation processes occurring. Here, both mass transport and the association process are neglected, and the biphasic dissociation curve are crudely modeled as two-independent sites, where occupancy at each site follows independent 1:1 binding and was fit to the analytic two-site model given in *Eqn (S7)*.

$$\text{Total Response } \mathbf{R_{(t)}} = \mathbf{R1}.\text{Exp}(-k_{d1}{*}t) + \mathbf{R2}.\text{Exp}(-k_{d2}{*}t) \quad \textit{Eqn (S7)}$$

Where **R1** and **R2** are the saturation responses for each binding site and k_{d1} and k_{d2} are the apparent dissociation rate constants.

Section 1B - Binding of PMBN to whole cells and OMV

It is difficult to isolate the formation of transient PMBL complexes due to rapid transition to long-lived cPMB clusters. However, PMBN is identical to PMB, other than the absence of an acyl chain associated with membrane interactions, and is therefore an ideal surrogate to investigate the initial transient interactions with LPS without the complexity of lipophilic membrane interactions[4]. For PMBN, it was possible to run replicates in a multicycle injection format because bound PMBN completely dissociated within a few minutes allowing multiple binding/unbinding cycles to be performed as needed in quick succession.

Binding of PMBN was tested on separate SPR sensing surfaces pre-coated with either *E. coli* cells or OMVs (**SI Fig. S12A** and **S12B**) as described in the main text and methods related to **Fig. 3**. This data is shown again in **SI Fig. S12** in triplicate in panels (**S12A**) and (**S12B**). The averaged binding affinity (K_D) and mass transport constants (k_t) over the three replicate model fits are shown

in panel (**S12C**). Each replicate was performed in series from top-to-bottom and a decrease in LPS binding activity was observed indicating a slow loss of LPS from these surfaces over time.

The kinetic curvature in the data was fully dependent on $\mathbf{R_{max}}$ indicating fully transport limited binding. Such binding is rarely observed, as it requires a very high reaction flux Lr = k_{on}.**[LPS]** but this is unavoidable here due to the combination of rapid electrostatically dominated binding of PMs to densely packed LPS surfaces. In this regime, a concentration gradient in PMBN develops at the sensing surface upon the start of an injection, and decays at the end, resulting in transport-driven kinetic curvature in the sensorgrams. In this regime, the observed kinetics vary as a function of LPS density rather the association rate constant of PMBN to LPS and, therefore, must be fit to an ordinary differential equation of the form shown in *Eqn (S3)*, where the binding rate constants are replaced by the affinity constant (K_D). The analysis shows that binding of PMBN to *E. coli* cells and OMVs produced similar affinity constants (**SI Fig. S12A-S12C**). The diffusion boundary model returns both K_D and k_t with high confidence, as indicated by the low standard errors associated with each returned parameter, despite the absence of any influence of binding kinetics.

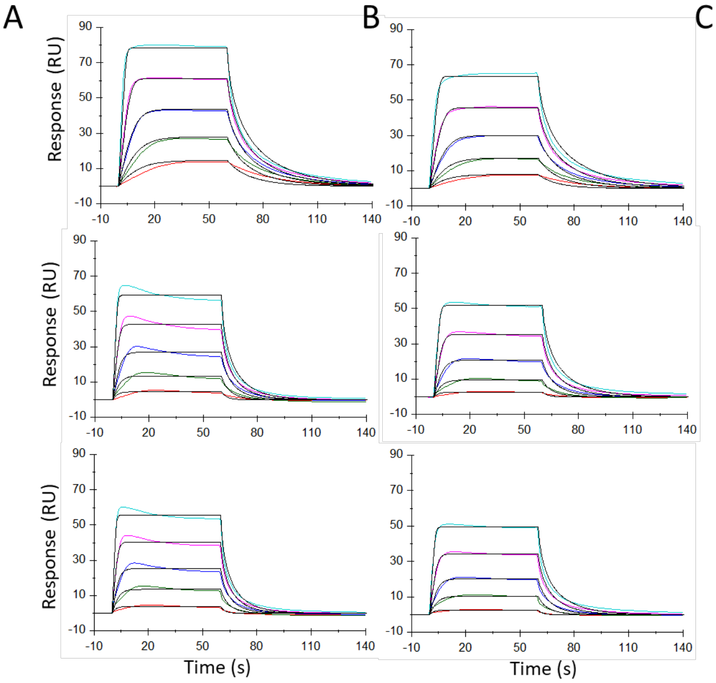

C

	K_D (nM)	$k_t \times 10^6$ (RU/Ms)
Cells	922 (± 0.08)	8.55 (± 0.08)
OMVs	1024 (± 0.11)	4.24 (± 0.03)

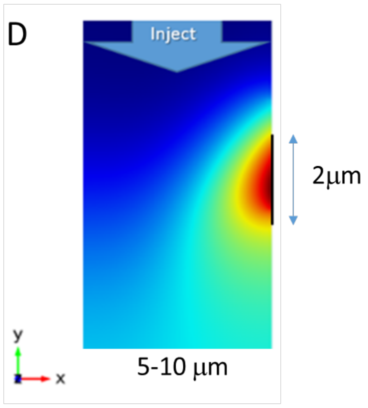

SI Fig. S12. Estimating affinity and transport constants from SPR binding curves from fitting a 1:1 kinetic boundary model (*Eqn (3)*) to binding of PMBN to (**A**) whole cells and (**B**) OMVs. (**C**) Fitted parameters returned from the fit in (A) and (B). (**D**) Numerical simulation of a flow capillary showing a mass transport boundary layer developing along an LPS-rich region corresponding to the length of a single *E. coli* with the color gradient representing **PMBN**, where highest depletion is represented in red and unchanged in dark blue. The average χ^2 was 1.17 RU^2 for fitted curves. Note that the fitted curves for cells and OMVs on the top row of (A) and (B) are shown in the main text (**Fig. 3A** and **3B**).

The fitted curves show dose-dependent kinetic curvature related to the development and decay of the mass transport-limited boundary layer. Inside this boundary PMB-binding kinetics proceeds rapidly, yet the curves reflect the slower kinetics of mass transport. This causes all points on these curves to represent pseudo-steady-state binding of PMBN, where the apparent time-dependence

of the curves emerges from the occupancy term 1/(**R$_{max}$**/**R$_t$**-1), which is independent of binding kinetics. The diffusion boundary model returns both K_D and k_t with high confidence as indicated by the low SE values. k_t is lower than would be expected, which is likely due to the three-dimensional topography of the bound cells and OMVs, which may be expected to contribute to added mass transport resistance[5]. It is straightforward to lower mass transport resistance by reducing the number of cells or OMVs bound to the SPR surface. However, using Comsol simulations (**SI Fig. S13**) we show that reducing the SPR flow cell to a 5 μm thick capillary increases k_t (i.e., decreases transport resistance) by almost 60-fold yet we still observe full mass transport limitation. This was expected because LPS remains densely packed on the surface and k_{off} for the interaction would need to be < 0.1 s^{-1} to allow kinetic binding to become partially limiting.

Section 1C - Single Cell Simulation

We used numerical simulation to visualize the development of the mass transport boundary layer and demonstrate that full mass transport limitation is expected even when PMBN binds discrete *E. coli* cells, as occurs *in vivo*. A single *E. coli* cell is approximated as a 2 μm long LPS-coated strip on the wall of a two-dimensional capillary (see **SI Fig S12D**). PMBN binding to LPS at the sensing strip was defined by *Eqn (1)* and the effects of mass transport limitation then arise from the finite element method where the advection-diffusion equations are solved in time-stepping mode throughout all 4230 elements meshed over the capillary area to produce realistic spatial concentration gradients as shown in **SI Fig. S12D** and the accumulation of complexes is then converted from moles/m^2 to response units and fitted to *Eqn (S3)* as shown in **SI Fig S13**.

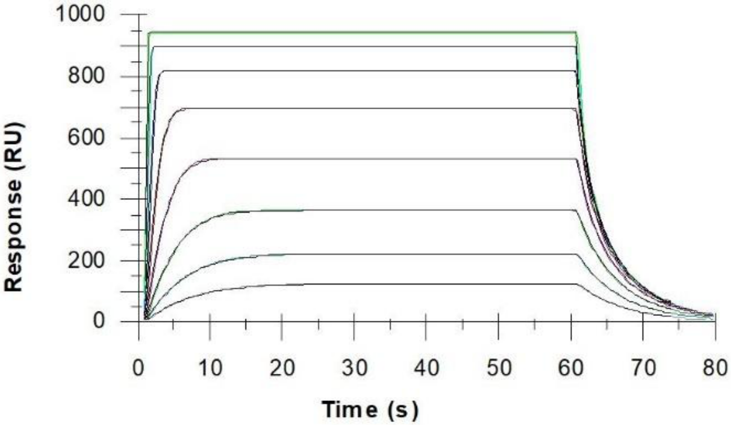

SI Fig. S13. Simulated binding of PMBN to an LPS-coated surface fitted to *Eqn. S3*. The LPS coating region (shown in **SI Fig. S12D**) is at an LPS coating density matching an *E. coli* cell surface (i.e., 0.77 ng/mm^2, equivalent to 770 RU). The simulation assumed transient kinetic constants with k_{on} =1x10^9 $M^{-1}s^{-2}$, k_{off} = 270 s^{-1}, a flow velocity 1 mm/s, and serial doubling dilutions of PMBN from 5 μM to 38 nM. *Eqn (S3)* was fit to the data returning the expected K_D as (270 ± 0.14) μM, while the apparent kinetic curvature specified an estimated k_t = 5.02 (± 0.002) x10^8 RU/Ms, which is consistent with the 2D k_t of 5.3 x10^8 RU/Ms estimated from theory. Diffusion of PMBN was assumed to be 4.08x10^{-10} m^2/s, matching that of the Comsol simulation[6].

In SPR the signal is averaged over a large surface area (~1 mm^2) containing a large number of bound cells or OMVs. Hence, mass transport is slowed due to the high local concentration which will overestimate the effect of mass transport on cells *in vivo* unless cells were to cluster. A numerical model representing single *E. coli* cell adsorbed on the inner wall of a 5 μm capillary was used to generate simulated binding curves analogous to our SPR experiment by solving the master equations of advection and diffusion using finite element analysis. A two-dimensional flow channel with a 2 μm reaction surface was simulated using the kinetic parameter values specified

in **SI Fig S13**. PMBN binding to the LPS at the strip was assumed to follow a 1:1 binding model and was modeled using a full set of ODEs that fully accounted for complex formation and depletion of both reactants. The effects of mass transport limitation arise from solving the advection-diffusion equations in time-stepping mode through the space elements of the discretized flow cell domain. This simulation allows analyte gradients to form mimicking the reaction at sensing surfaces when PMBN is consumed faster than it can be replenished via convection and diffusion. Therefore, this simulation allows *Eqn. (S3)* to be validated beyond the practical limitations imposed in real SPR experiments. The Comsol curve set was fit to *Eqn. S3* while solving for k_t and K_D, resulting in a superimposable fit. The k_t value returned in the fit (5.02×10^8 RU/Ms) was in agreement with that (5.3×10^8 RU/Ms) estimated from analytical solution for transport in the SPR flow cell[7]. As expected, the fundamental rate constants used in the Comsol simulation could not be recovered when fit to a conventional two-compartment model (*Eqn (S2)*) but k_t and K_D were accurately recovered using the boundary layer model (*Eqn (S3)*). The fitted simulation also shows that binding will remain dominated by transport resistance in vivo despite a ~60-fold higher k_t relative to our SPR experiments.

Configuring the Numerical Simulation

The simulation was performed using Comsol multiphysics 5.1 (COMSOL AB, Tegnérgatan 23, SE-111 40, Stockholm, Sweden). Parameter values employed in the numerical simulation are given in **SI Fig. S13**. A single whole *E. coli* is approximated as a 2 μm long LPS-coated strip on the wall of a two-dimensional capillary (see **SI Fig S12D**). PMBN binding to LPS at the sensing strip was defined by *Eqn (1)* and the effects of mass transport were simulated in time-stepping mode throughout all 4230 elements meshed over the capillary area to produce realistic spatial concentration gradients as shown in **SI Fig. S12D.** The accumulation of complexes is then converted from moles/m^3 to response units and fitted to *Eqn (S3),* as shown in **SI Fig S13**. The incompressible form of the Navier-Stokes equation was used to solve the two-dimensional velocity profile through the channel, assuming steady-state at constant flow rate and at atmospheric pressure. The velocity at the walls $u_{wall} = 0$, the inlet velocity $u_{inlet} = 1$ mm s^{-1} then solving for the velocity vector field over the full domain assuming $\nabla.u = 0$ and $\rho u \cdot \nabla u = -\nabla p + \mu \nabla^2 u$, where ρ is the density, p is the pressure and μ is the dynamic viscosity. The flow velocity vector field was coupled to the steady-state advection/diffusion equation for a dilute species to solve for the analyte concentration field in the bulk flow. Where $\nabla.(-D\Delta_C) + u.\Delta c = R$ and D is the diffusion coefficient of PMBN, c is its concentration and R is a binding reaction term. Initially the analyte concentration in the microchannel $c = 0$. At the inlet the initial analyte concentration profile along the microchannel height is assumed constant and the start and end of each injection is defined by multiplying the concentration by a time-dependent rectangular step function with lower-to-upper limits from 0-1. Target is bound to the wall of the flow cell requiring a surface reaction at the wall. The associated surface reactions were modeled by a *surface molar flux*, $N_{t,i}$ according to $N_t = - D_{s,i}\Delta_t c_{s,i}$, where $D_{s,i}$ is the surface diffusion coefficient for species *i* at concentration $c_{s,i}$. The governing equation for the surface reaction is $\Delta_t = (- D_{s,i}\Delta_t c_{s,i}) = R_s$, where R_s is the surface flux balance (mol m^{-2}) with $D_s = 0$. The reversible binding reaction at the surface was given by a phenomenological 1:1 pseudo-first order model, as in *Eqn (S1)*, but here biosensor response is replaced by the concentrations of each species where $(\delta c_{si} / \delta t) = k_{on} c(c_{max} - c_s) - k_{off} c_s$. The binding flux of the analyte to the target-coated sensing surface was balanced by a coupled flux loss from the bulk liquid. The time-dependent change in analyte accumulation was found from a surface flux balance at the sensing surface. The accumulation of the affinity complex at the planar surface was expressed in terms of an equivalent biosensor response, where 1 RU is assumed equal to 1.0 pg/mm^2. The simulation results (**SI Fig. S13**) were exported into Biaevaluation V4.1 (Cytiva 100 Results Wy Marlborough, MA 01752) and fit to *Eqn (S3)*.

Section 1D - Multipoint Chaser Binding

Repeated PMBN injections over a sensing surface that has been pre-saturated with PMB were exploited to report loss in PMB occupancy over an extended time period. Progressive dissociation of PMB causes a corresponding increase in transient PMBN binding since PMBN binding is proportional to free LPS, which varies inversely with PMB dissociation. The resulting time-course measurements allow PMB dissociation to be plotted (**Fig. 3H**). This chaser technique provides an indirect measure of occupancy that is free of long-term drift and other interferences that sometimes complicate the measurement of tightly bound complexes by direct SPR. A simple two-site dissociation model (*Eqn (S7)*) was fit to the data. The analysis revealed two apparent populations of PMB clusters driven by moderate and extremely low dissociation, respectively.

Section 1E - Binding of PMB to an LPS-coated sensing surface pre-saturated with polymyxin B

Pure LPS vesicles were coated onto two planar sensing surfaces in order to study the interaction with polymyxin B after initial pre-saturation with polymyxin B. The use of pure LPS-vesicles isolated the contribution of LPS to polymyxin B retention since outer membrane proteins and membrane phospholipids are absent. Following from multipoint chaser binding (described in **SI Section 1D)**, we repeated serial polymyxin B injections after pre-saturation of the LPS surface with polymyxin B thereby resolving the reversible transient binding component more clearly (**SI Fig. S14**). This transient binding profile resembles PMBN binding, other than returning a higher affinity (K_D ~127 nM, see **SI Section 1D** and **SI Fig. S14**) relative to PMBN. The higher affinity relative to PMBN likely results from lipophilic interactions that do not result in full membrane insertion but do augment the stabilizing electrostatic interactions within the LPS affinity network. These interactions likely cause bound polymyxins to rebind repeatedly before escaping via diffusive transport[8].

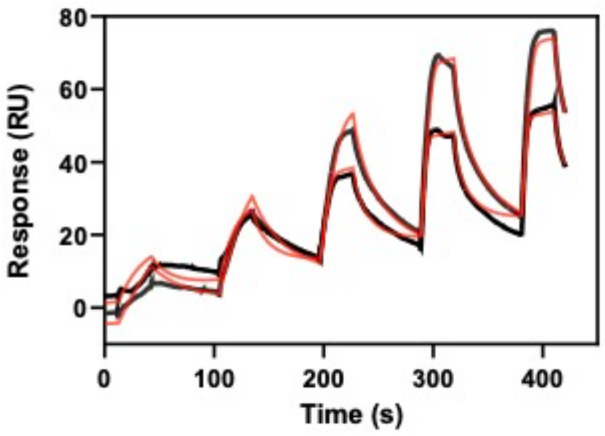

SI Fig. S14. Binding of polymyxin B to two LPS-coated sensing surfaces of different LPS density where each was pre-saturated with polymyxin B. Serial doubling concentrations of polymyxin B up to a top concentration of 625 nM were performed. The curves were fit to *Eqn. (S3)* returning an approximate affinity constant of 126.9 (± 1.4) nM and a moderate χ^2 = 5.7 RU. All parameters other than R_{max} were constrained globally, and a response offset term was introduced to negate the slight baseline offset between each curve. These data were obtained by duplicate injection of polymyxin B using SCK injection mode over LPS-coated sensing surfaces that had been pre-saturated with polymyxin B, where this second injection series covered a five-step serial-doubling dilution range from 625nM.

Section 1F – Polymyxin B-LPS Binding Valency

Pure LPS was coated onto a planar C1-chip surface in order to study the interaction with PMBN and polymyxin B in the absence of both outer membrane proteins and phospholipids (**SI Fig. S15**). Note that **SI Fig. S15A-S15B** and **S15D-S15E** contain data shown in **Fig. 3C** but here a duplicate set of data is added for a second LPS-coated surface. The polymyxin B curve sets shown in **SI Fig. S15C** are the data in **Fig. 3D**. The associated methods are therefore as given in the Materials and Methods section. **SI Fig. S15A** and **S15B** show SPR curves for PMBN recorded at different LPS densities and curves in **SI Fig. S15D** and **S15E** are replicates performed after saturating the same sensing surfaces with PMB (**SI Fig. S15C**). This sequence of injections was performed in order to determine residual PMBN binding activity to LPS after pre-saturation with polymyxin B. The analysis revealed how much LPS was consumed through persistent interactions with polymyxin B immediately after saturation with polymyxin B. A large fraction of polymyxin B remained associated with LPS immediately after the polymyxin B exposure ended and competitively inhibits PMBN binding at these sites (**SI Fig S15A-B and D-E**). **SI Fig S15D** and **S15E** also show an increase in boundary layer kinetics and a 2-fold loss in both affinity and PMBN binding capacity relative to **SI Fig S15A-B** and are consistent with a loss in available LPS. When normalized by the molecular weight ratio of polymyxin B (PMB):PMBN, the R_{max} values for PMB (407 RU) and PMBN (415 RU) indicate ~40% consumption of available LPS. A comparison of PMBN binding curves, normalized with respect to response, is shown in **SI Fig. S15F**, and shows accelerated PMBN kinetics after pre-saturation with polymyxin B and is consistent with the dominance of mass transport limitation and is well defined by the diffusion boundary model (*Eqn (S3)*).

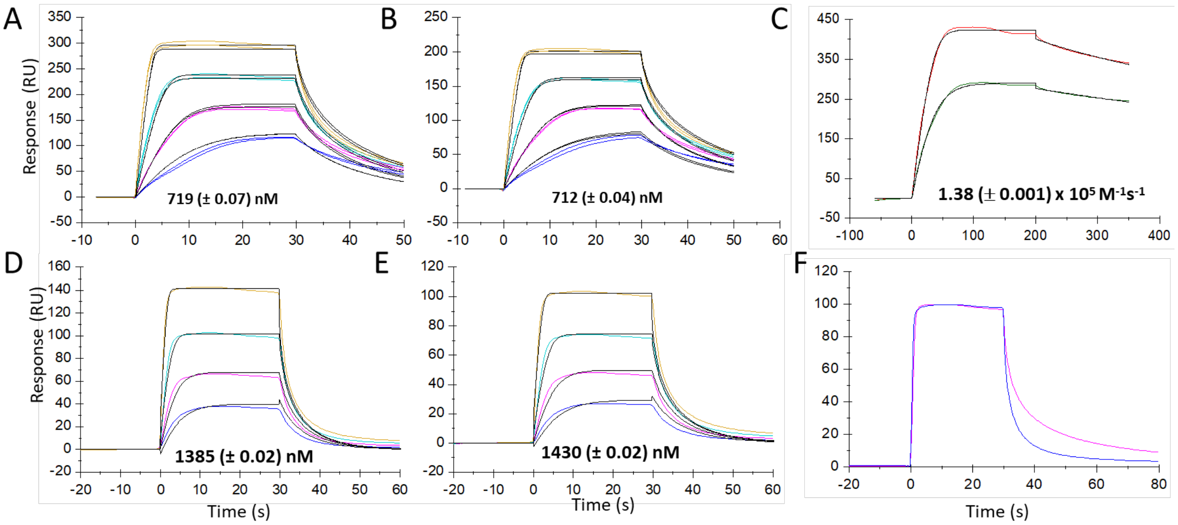

SI Fig. S15. SPR curve sets for sequential exposure of LPS-vesicle surfaces to PMBN (A and B) then polymyxin B (PMB, C), and, finally, PMBN (D and E). (**A** and **B**) Duplicate injections of PMBN were performed over a serial doubling dilution range at two LPS-coating densities and fit to *Eqn (S3).* (**C**) PMB (1 μM) saturation curves performed over the respective LPS surfaces immediately after completion of the initial PMBN curve sets (A and B) and the resulting curves were fit to *Eqn (S1)*. (**D** and **E**) Repeat of binding curves of (A and B) performed 20 minutes after saturation of both surfaces with PMB in (C) and fit to *Eqn (S3)*. (**F**) PMBN (5 μM) binding curves recorded before (pink curve) and after (blue curve) PMB saturation, where both curves have been normalized with respect to response to allow comparison of the dissociation profiles.

Section 1G - Calculation of Binding Stoichiometry

Pure LPS vesicles bound to a polymyxin B-derivatized sensing surface were used to generate the PMBN and PMB binding data shown in **Fig. 3A-3D**. Purified LPS isolated from a deep rough Rd *E. coli* strain (F583, Sigma L6893), which is a minimal LPS molecule comprised of lipid A decorated with 2 2-keto-3-deoxyoctonic acids and 2 L-glycerol-D-mannoheptose sugars and has an average molecular weight of 1.7-1.8 kDa, was used and denoted as rh-LPS for the following calculations. The calculated physical properties of lipid A, PMB, and PMBN are provided in the table below (MW and RI computed using chemspider.com). The calculated MW of lipid A is close to the MW reported for rh-LPS, as expected due to the minimal core oligosaccharide present on this species and was used for calculations. Using the SPR response and MWs of each species, we show that the binding stoichiometry of either PMB or PMBN to LPS is approximately 1:2. Calculation requires normalization with respect to MW and refractive index (RI).

molecule	MW	RI	Refractive Index Ratio
**Lipid A**	1798	1.521	1
**PMB**	1203	1.592	1.047
**PMBN**	963	1.608	1.057

The average stoichiometry of PMB occupancy can be estimated by SPR from these experiment as follows:

- Total PMB bound at saturation = 475 RU
- Total LPS coated = 1500 RU
- MW of rh-LPS = 1798 Da, MW of PMB = 1203 Da
- Respective response ratio = (1798 Da/1203 Da * 1.047) = 1.42
- Expected PMB binding response assuming 1:1 stoichiometry = 1500 RU/1.42 = 1051 RU
- Therefore, actual binding stoichiometry = (1051 RU/475 RU) = 2.2, thus LPS:PMB = 2.2:1

This calculation was also performed for PMBN binding to the same surface.

- Total PMBN bound at saturation = 414 RU.
- Total LPS coated = 1500 RU.
- MW of rh-LPS = 1798 Da, MW of PMBN = 963 Da
- Response ratio = (1798 Da/963 Da * 1.057) = 1.77
- Thus, the expected PMBN binding response assuming 1:1 stoichiometry = 1500 RU/1.77 = 847 RU
- Therefore, actual binding stoichiometry = (847 RU/414 RU) = 2, thus LPS:PMBN = 2:1

Section 1H – Non-specific binding of PMBN and polymyxin B

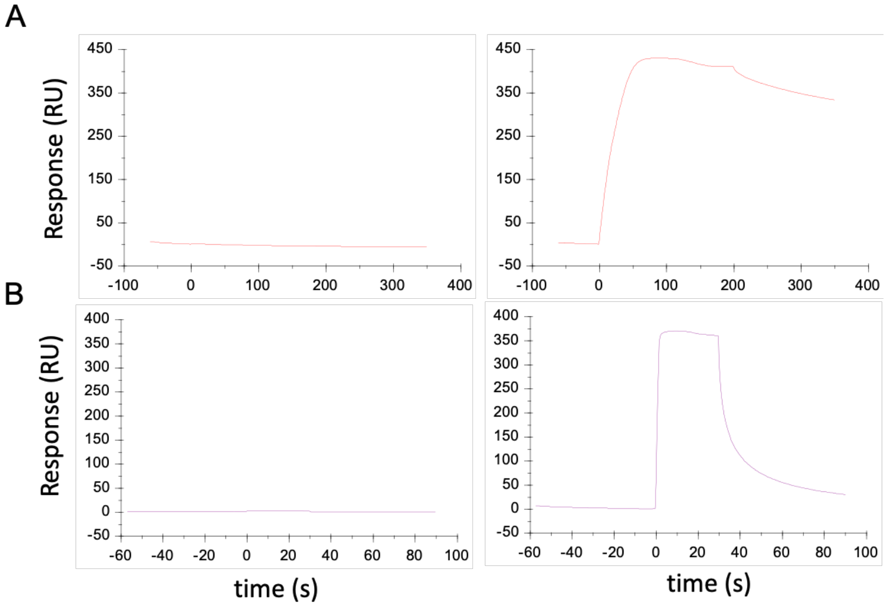

SI Figure S16. Evaluation of non-specific binding. Non-specific binding and specific binding to a C1 sensor chip without reference curve subtraction. (**A**) 1 µM PMB was injected (50 mL/min for 200s) over two sensing surfaces where one channel remained uncoated (left panel) and LPS has been pre-coated (right panel) onto the other. From the raw response curves (no referencing) we observed a response of -6 RU or the uncoated surface and a response of 428 RU for the LPS-coated surface. (**B**) The experiment in (A) was repeated where 10 µM PMBN was injected (50 mL/min for 30s). The raw response curves gave a response of -0.4 RU for the uncoated surface and a response of 368 RU for the LPS-coated surface.

Section 1I - Assumptions derived from mode-of-action experiments (sections 1B-1H)

1. PMBN binds transiently under full mass transport limitation with effective K_D in the nM to low µM range. PMB exhibits similar transient binding, but a large fraction of these transient complexes become strongly retained suggesting a multistate mechanism.

2. Initial transient binding is driven by complementary electrostatics and as is competitive with binding of divalent metal ions. We observed that binding is attenuated in high salt or with high concentrations of divalent cations as others have also demonstrated.

3. Full mass transport limitation was observed, preventing estimation of the fundamental binding rate constants for formation of PMBL, but implies that these rates are highly transient, and this is expected to hold *in vivo*.

4. Pure LPS bilayers were sufficient to recapitulate binding of both PMB and PMBN indicating that OMPs and other membrane components are not essential for prolonged retention of PMB, but this does not eliminate the possibility that other processes such as blebbing may be coupled to other outer membrane components.

5. The consumption of LPS in forming stable PMB states, the recovery of free LPS over time, and the concomitant accumulation of strongly retained PMB implies that the most stable bound PMB state is not in complex with LPS.

6. The observed dissociation process indicates that the majority of tightly bound PMB is essentially irreversibly retained at practical time scales, which is consistent with the proposed cluster model.

7. The accumulation of stable PMB was observed for whole cells, OMVs, and pure LPS films, and in all cases approaches a saturation limit that is proportional to the total LPS concentration on the surface. LPS represents approximately 50% of the outer membrane of whole cells and yet we show that the long-lived component is no longer in association with LPS. Mass conservation implies that this extreme additional mass must be partitioning into the outer membrane in the absence of affinity interactions and, therefore, requires stretching of the outer membrane. This is in good agreement with the known action of polymyxins in weakening the LPS network by competing with stabilizing divalent cations.

8. Experiments with resistant forms of LPS show minimal accumulation of stable PMB states while transient binding to LPS is retained. These resistance mutations inhibit membrane stretching by stabilizing the LPS affinity network, which in turn supports a mechanism where the capacity of the membrane to stretch is assumed to define the upper limit for PMB clusters to accumulate.

9. Clustering mediated by self-interactions and possibly in combination with phase separation could create the stability of all bound PMB states beyond the initial transient PMBL complex. Pure LPS-coated surfaces are sufficient for stable PMB accumulation where stable states exist without being in complex with LPS, pointing to phase separation as a probable driving force for retention of bound PMB.

10. Overall, these insights imply a multistate model, where initial electrostatic binding of PMB displaces stabilizing divalent cations, which enables membrane stretching and in turn facilitates accumulation of stable PMB, which tends to form clusters due to the influence of lipophilic interactions. This does not preclude other models consistent with our kinetic measurements but will require additional structural insight at resolutions not yet achieved.

Section 2

Section 2A - Formulation of three-state model for fitting SPR data

The three-state model (**Fig. 4**) was formulated as a set of coupled ordinary differential equations (ODEs), with eight binding rate constants ($k_{1\text{-}8}$) governing the rates of interchange of each species. Forward rate constants k_7 and k_9 were eliminated from the equation set as formation of transient species tPMB and LL are irreversible.

Analysis constants

Time at Injection 1 start = 0 s
Sample contact time = 30 s
Dissociation time interval = 35 s
Mass transport rate = k_t = 46.5 1/s

Initial Species Concentrations

Coupled ODEs of Three-State Model

$$d[L]/dt = -(k_1.[PMB].[L] - k_2.[PMBL])+2*k_{10}.[LL] \quad (S8)$$

$$d[PMB]/dt = -(k_1.[PMB].[L] - k_2.[PMBL]) + k_t'.([PMBi]_{(t)} - [PMB]) \quad (S9)$$

$$d[PMBL]/dt = (k_1.[PMB]*[L] - k_2.[PMBL])-(k_3.[PMBL].[n] - k_4.[nPMBL]) \quad (S10)$$

$$d[n]/dt = -(k_3.[PMBL].[n] - k_4.[nPMBL]) \quad (S11)$$

$$d[nPMBL]/dt = (k_3.[PMBL].[n] - k_4.[nPMBL])-2.(k_5.[nPMBL].[nPMBL] - k_6.[tPMB].[LL]) \quad (S12)$$

$$d[tPMB]/dt = (k_5.[nPMBL].[nPMBL] - k_6.[tPMB].[LL]) - k_8.[tPMB] \quad (S13)$$

$$d[cPMB]/dt = 2*k_8.[tPMB] \quad (S14)$$

$$d[LL]/dt = (k_5.[nPMBL].[nPMBL] - k_6.[tPMB].[LL]) - k_{10}.[LL] \quad (S15)$$

The initial concentrations of each species were as follows

$$[PMB]_{t=0} = [PMBL]_{t=0} = [nPMBL]_{t=0} = [tPMB]_{t=0} = [LL]_{t=0} = [cPMB]_{t=0} = 0$$
$$[L]_{t=0} = [n]_{t=0} = 1.56 \times 10^{-4} \text{ (units, M)}$$

k_t' (units, m/s) was already defined in SI Section 1A. $[PMBi]_{(t)}$ is the injected concentration profile and $[PMB]_t$ is the concentration profile at the sensing surface. $[PMBi]_{(t)}$ follows a serial-doubling concentration of injected analyte, with a concentration profile defined by a serial step function, where each injection step represents a discrete concentration followed by a dissociation step without injected analyte and this is repeated for each concentration in the SCK dosing series. Kintek Explorer V9.5 was used to build and fit the three-state model. This program employs its own numerical integrator[9], which reports the change in concentration of each species over time. The SPR responses for concentrations of each accumulating PMB-species estimated from *Eqn (8 - 15)* are summed over time in *Eqn (1),* repeated here.

$$Response_{(t)} = ([PMBL]_t+[nPMBL]_t+2*[tPMB]_t+m.[cPMB]_t).MW.G. \quad \textit{Eqn (1)}$$

The concentration of $[tPMB]_t$ is multiplied by two to account for its additional mass, being composed of two PMB molecules. Insertion of PMB into the acyl-LPS matrix to form cPMB is associated with changes in the phase state of LPS[10] and is also likely associated with redistribution of mass towards the sensing surface[11]. These changes will manifest as an increase in sensitivity of the SPR binding response when clusters are formed and are included in *Eqn (1)*

using a sensitivity coefficient (m). MW is the molecular weight of the injected analyte, in this case PMB (1203.48 Da). The constant, G, is a unit conversion factor that converts protein concentration (g/L) to response (RU) and is typically 100 (units, RU.L/g). Biacore SPR systems are calibrated such that 1 RU is equivalent to a change of 1×10^{-6} refractive index units (RIU), which is equivalent to a 2D concentration of 1 pg/mm^2 protein when the mass is distributed uniformly within a 100 nm hydrogel. The average height for OMVs bound to the surface is assumed to follow solution phase size measurements of approximately 100 nm and therefore G is assumed to apply as a reasonable approximation. In practice, the value was optimized within ± 6% when model fitting to allow for variations in OMV capture yield and spatial distribution effects. Assuming 1:1 binding stoichiometry, an OMV where LPS is fully saturated with PMB will contain approximately 0.77 ng/mm^2 PMB, which is equivalent to ~770 RU. Therefore, a PMB binding capacity of 30 RU on an OMV-coated surface, shown in **Fig. 5,** represents just 4% of an equivalent full OMV monolayer, or approximately 156 µM LPS when expressed as a 3D concentration.

Extreme depletion of PMB occurs when the surface reaction flux coefficient k_1.[L] is high relative to k_t' preventing estimation of the transient rate constants (k_1 and k_2) associated with PMBL formation. Therefore, the rate equation for the diffusion boundary model (*Eqn (S3)*) was substituted into *Eqn. (S10)*, effectively replacing k_1 and k_2 with K_D as shown in *Eqn (S16)* below.

$$d(PMBL)/dt = (k_t'.[PMBi]_{(t)} - k_t'.K_D.([PMB]_{t=0}/([PMB]_{t=0}-[PMB])-1)^{-1}-(k_3.[PMBL].[n]-k_4.[nPMBL]) \quad \textit{Eqn (S16)}$$

When performing SPR curve fitting, it is common to express the mass transport coefficient in terms of SPR response, where k_t (units, RU/Ms) = $k_t'.100.MW_{PMB}.R_{max}$. The rate constant k_3 for PMBL insertion into an LPS-associated membrane insertion site (n) equals the rate constant k_6 governing recovery of nPMBL from the transition state (tPMB). Both processes require membrane insertion and k_3 is the rate at which membrane insertion sites become available. Similarly, the rate constant k_4 governs dissociation of nPMBL from membrane insertion sites (n) and equals the rate constant k_{10} (*Eqn (S8)*) for liberation of LPS during decay of the transition complex (shown as k_4 (blue) in **Fig. 4**). This rate constant defines the stability of membrane insertion for both nPMBL and tPMB. The stability of the model fit was improved by setting k_6 and k_{10} equal to k_3 and k_4, respectively. This reduced the number of binding rate constants to be fit to just four. The numbering of rate constants remains as in *Eqn (S8 - S15)* except for the repeating rate constants mentioned above, which retain the numbering of the first instance.

Section 2B - An Interpretation of the Three-State Model

The complex mode-of-action of PMB has been linked to its amphiphilic structure where relatively weak electrostatic interactions with LPS eventually results in tightly bound PMB and is known to be associated with lipophilic interactions[12]. Precisely how this occurs has remained speculative because of a paucity of kinetic methods to study the associated processes on cell surfaces. Here we address this deficit in understanding by developing a set of SPR assay formats to test PMB binding (and analogs) to whole cells, OMVs and LPS that are coated onto planar SPR sensing surfaces. This enabled a three-state kinetic model to be derived from the SPR data, which provided a kinetic mechanism for LPS-catalyzed PMB self-promoted uptake. The fraction of bound PMB in each of the three states is measurable since the SPR response detects the mass change and kinetic curvature associated with formation and decay of each state.

Initially, long-range electrostatic pre-concentration of PMB promotes rapid formation of electrostatic interactions with the phosphate groups of each LPS molecule, which are stronger at the lipid A core. The extremely high density of LPS results in accumulation of a high density of PMB as these interact to form 2:1 LPS:PMB complexes with near saturation for PMB in the low µM to nM range. These transient LPS interactions also competitively displace the divalent cations

required to maintain the integrity of the LPS layer. Loss of LPS layer stability renders the membrane vulnerable to stretching which provides potential PMB insertion sites. The LPS bound state positions PMB favorably for integration of its lipophilic anchor into the acyl matrix of LPS. This LPS-catalyzed membrane integration of PMB allows lipophilic interactions to dominate over tPMB-LPS affinity interactions leading to PMB clustering with liberation of LPS, which can continue through repeated catalytic cycles. This process leads to high local concentrations of clustered PMB in the outer membrane likely forming PMB aggregates, cPMB, that penetrate into the cytosol which would be consistent with the self-promoted uptake model.

Section 2C - Model Optimization and Curve Fitting
The boundary layer model (*Eqn (S3)*) provided good estimates for reversible binding of PMBN to LPS (**Fig. 3A** and **3B**). Briefly, *Eqn (S3)* was fit to this data using Biaevaluation (leading SPR-analysis software) where the initial guess for K_{D1} was obtained by first fitting an affinity model to the steady-state regions of these same data sets. k_t was calculated from theory [6] and a global fit returned values that were then used as starting values for the three-state model fit of polymyxin B binding to LPS (**Fig. 5**). In modeling polymyxin B binding to resistant-OMVs kinetic constants for state 3 tended to zero while the other fitted parameters remained resolved. Thus, state 3 was eliminated from the model, effectively by the fitting algorithm, resulting in the fit in **Fig. 5B**. The three-state model was then fit to the polymyxin B data where initial parameters for k_t, K_{D1}, k_3, and k_4 were taken from the PMBN fit. The simulation did not resemble the data set and therefore the initial values for the added kinetic rate constants associated with the transition state and state 3 were iterated manually. This was performed using Kintek's dynamic simulation function where one can drag the value of any parameter over a wide range of values in just a few seconds and the simulation, which is overlaid on the actual curves to be fit, responds in real time (no apparent delay). In this way the observed curvature and scaling of the binding curves to be fit are roughly reproduced in the simulated curves. Using the initial values from manual dynamic simluation, a global fit showed some instability due to the higher number of parameters being estimated. Therefore, K_{D1} was held constant at the value taken from the affinity fit in **SI Fig. S14**. The fit was then repeated, and it was noted that k_3 and k_4 were repeating as k_6 and k_{10}, respectively, and this removed another two rate constants leaving just three binding constants and k_t to be estimated. A fit with these constraints produced a high-quality fit as shown in **Fig. 5A**, where initial values were found by manual dynamic simulation and repeating this fitting process multiple times had no significant effect on the results.

Section 2D - Curve fitting and plotting
Curve fitting programs enable fitting of binding interaction data to interaction models by nonlinear regression, and the associated statistical methods to confirm goodness of fit and confidence in parameter estimates are well established. The goodness of fit between a model curve and an experimental curve is best described by χ^2 when the number of data points is high and by a regression coefficient R^2 when the number of values is low. χ^2 is the square of the averaged residual response difference and approaches the baseline noise for the best fits. Statistical parameters such as the standard error of the fit (SE) associated with a given parameter returned in the fit are used to report confidence in fitted parameter estimates. This form of standard error is not related to the standard error of the mean for replicate values of a set of measurements and has a different interpretation. The SE of the fit is based on the covariance matrix for the fitted parameters and can be considered as a measure of the information content of the data and specifies the degree to which the curves define the parameter value from the fit and its confidence limits. Generally, we assume that values that approach 10% of the parameter value indicate low confidence and often indicate overfitting. However, when fitting complex models it's important to understand correlations between fitted parameters and the 2D FitSpace analysis[9] provides a reliable "brute force" algorithm to measure the "true" confidence limits and parameter correlations.

Application of FitSpace analysis showed that all parameters are well constrained validating that parameter values returned from the fit are unique as opposed to being the product of over-fitting. Kintek Explorer provides a simple reaction scheme interface to define models and automatically generates the ODEs (*Eqn (S8 - S16)*) that are integrated numerically and fit through non-linear regression. Numerical integration is also used in Biaevaluation 4.1 and Biacore S200 evaluation software (both from Cytiva 100 Results Wy Marlborough, MA 01752) where models are entered as ODEs or simple analytic solutions. With the exception of **Fig. 5**, Biaevaluation allowed all other rate equation-based models to be fit and plotted (**Fig. 3A-3G** and **SI Fig. S12-S15**). However, Graphpad prism V9 was employed to fit some analytic models (**Fig. 3H**) and to produce publication quality plots of fitted binding curves (**Fig. 1**, **2**, **5**, and **SI Fig. S14**). Microsoft Powerpoint was employed for image processing and Microsoft Excel was used for data reduction.

SUPPLEMENTAL METHODS

Binding and kinetic analysis of SPR-OMV assays

To determine the apparent- K_D of the reversible binding event of polymyxin B, single-cycle kinetics were performed as described in Materials and Methods. After OMVs were loaded, 5 µM polymyxin B was injected for 360 sec (40 µl/ml flow rate) followed by 120 sec dissociations to saturate the stable-binding population prior to the sample injections. The base-to-peak value of each trace was determined from double-referenced traces (reference 1: - OMVs/ + compound channel; reference 2: + OMVs/ - compound) exported from the Biacore S200 evaluation software into PRISM 9 software. The change in RU with each pulse was plotted over the concentration and the K_D determined by fitting a non-linear regression, one-site total function with background parameter set to 0 (GraphPad Prism version 9.3.1 for Mac, GraphPad Software, San Diego, California USA, www.graphpad.com). To determine the apparent K_D of nonapeptide and polymyxin B on resistant-OMVs the same approach was taken as described above but without any pre-saturation step prior to kinetic pulses.

To determine the residence time of the test compound with OMVs, the 'chaser method' was used because it accounts for drift that can occur in the system over long incubation times [13]. OMVs were loaded onto the chip as described above then 5 µM polymyxin B was injected using the 'low-sample consumption' setting for 300 sec (5 µl/ml flow rate), followed by a 240 sec dissociation time. A second dose of 5 µM polymyxin B was then injected for 60 sec (30 µl/ml) to assure saturation followed by a 2 h dissociation time prior to the 'chaser', a 60 sec pulse of 5 µM polymyxin B. The RUs of associated polymyxin B were determined 120 sec after the start of the dissociation. The change in RUs from before dosing to after the second dose, and the change in RUs from prior to the 'chaser' dose and 120 sec after the start of its dissociation were used to calculate the fraction occupancy of the material and the residence time/half-life as described [13].

Cryo-EM grid preparation and data collection

Wild-type and polymyxin-resistant OMVs (*pmrA*^{G53E}) were incubated with polymyxin B, PMBN, or an equal volume of buffer, as described for TEM imaging at a final ratio of 0.5:1 polymyxin B (or PMBN) to LPS for 20 min. To prepare samples for cryo-EM, 4 µL of each condition was applied to holey gold grids (UltrAuFoil 25 nm R 1.2/1.3; Quantifoil) which had been glow discharged for 20 sec with a Solarus plasma cleaner (Gatan). Grids were blotted and plunge-frozen by Vitrobot (ThermoFisher Scientific, Waltham, MA) at 4°C, 100% relative humidity, blot force 7, and a 3 sec blot time.

TEM data collection was performed using a Titan Krios G3i (ThermoFisher Scientific, Waltham, MA) operating at 300 kV equipped with a BioQuantum energy filter and a K3 Summit direct electron detector operating at a nominal magnification of 105,000x (0.838 Å/pixel), an energy slit width of 20 eV, defocus range -1 to -3 µm, 3-sec exposure time, 60 frames per movie, a total electron fluence of ~64 e/Å^2. Microscope and camera automation was accomplished by SerialEM[14], and image processing (motion correction & CTF estimation) was performed using CryoSparc[15].

Standard cryo-EM image processing software and methods were incompatible with the vesicle images, so a blinded visual scoring method for image analysis was used. Approximately 50 images from each sample and treatment condition that each contained at least one OMV (pooled in a single folder and blinded) were scored for number of vesicles touching (0-10+), and if the vesicle membranes appeared disrupted or abnormal. Sampling was not completely random as images of vesicles could not be obtained from areas with significant clumping. Images shown in

SI Fig. S2 were low-pass filtered and down-sampled to enhance contrast but not the bulk images used for quantification.

Lipid A extraction and mass spectrometry analysis
Lipid A was extracted from OMVs by using the method described for 5 mL culture volume of whole cells in[16] (Section 3.6) as follows. 50 µL (~50-75 µg) of the OMV preparation was extracted by single-phase Bligh-Dyer (chloroform:methanol:water at 1:2:0.8 v/v), vortexed, and incubated for 20 min in glass tubes. The sample was centrifuged for 20 min at 1500 x*g* and the supernatant was removed. The pellet was suspended in 1.8 mL hydrolysis buffer (50 mM sodium acetate, pH 4.5, 1% sodium dodecyl sulfate (SDS)), sonicated for 30 sec in a bath sonicator, boiled for 30 min, and cooled for 10 min. 2 mL of chloroform and 2 mL of methanol were added (final chloroform:methanol:aqueous at 2:2:1.8 v/v) and the sample was vortexed and centrifuged as before. The lower phase was retained and extracted again with 2 mL chloroform. Dried samples were brought up in 1 ml of 0.25% n-Dodecyl-B-D-Maltoside (DDM) detergent in water by heating to 42°C and bath-sonicating.

Lipid A LC-MS/MS analysis was performed on a Thermo qExactive orbitrap mass spectrometer with an electrospray ionization source in negative mode. The LC was a Thermo Ultimate 3000 and LC eluent was split between the qExactive MS and a Thermo charged aerosol detector (CAD). The LC separation was performed at 40 °C on a Phenomenex Luna 5 µm C8 100 Å, 50 x 2 mm column. A 15-min gradient utilized solvent A (10 mM Ammonium Acetate in H_2O) and solvent B (isopropyl alcohol:acetone:ethanol at 2:1:1). A linear gradient with the following proportions (v/v) of solvent B was applied: 0 – 5 minutes at 1%, 5 – 15 minutes at 99%, 15 – 20 minutes at 99%, 20 – 20.25 minutes at 1%, and 20.25 – 25 minutes at 1%. The LC eluent was diverted from the MS to waste for DDM elution 11.20 to 12.55 minutes. MS^- full scan data was acquired from *m/z* 1000 – 3700 and *m/z* 200 – 2200. The following source settings were used: spray voltage was 3400 V, capillary temperature was 320°C, sheath gas was 45, auxiliary gas was 10, and probe heater temp was 450°C. Relative quantitation between ratios of unmodified, single-modified, and double-modified lipid A were evaluated in MS^- mode extracted ion chromatograms. Heavy labeled standards for absolute quantitation and statistical analysis were not available. The extracted ion chromatogram peak areas were integrated and reported. The intensity of all integrated EIC peaks were within the linear range of the mass spectrometer. Lipid A analogues of methylene extensions were detected of the singly, doubly, and unmodified lipid A. Data analysis performed with Thermo Xcalibur Qual Browser 3.0.63 and full spectra are provided in Source Data file.

SPR assay methods development
Commercially available lipophilic chips are able to non-specifically bind liposomes and vesicles. Membrane vesicles (derived from Expi293 cells[17]**, SI Fig. S4A**) and *E. coli* OMVs (**SI Fig. S4B**) were immobilized on the surface of a lipophilic chip (LP, Xantex Bioanalytics full method description below). Lipophilic chips are not compatible with detergents however, which are necessary to reduce non-specific binding and loss of polymyxins to plastics[18]. The lipophilic chip surface also interacts with polymyxin B (**SI Fig. S4C and S4F** dashed line) which, in combination with the plastic-binding properties of polymyxins likely contributed to the inability to resolve interaction with OMVs (above interaction to the chip) at lower concentration using this method (**SI Fig. S4C**). To overcome these limitations, OMVs were immobilized via amine-coupled polymyxin B to the surface of a C1 chip (see materials and methods). OMVs were stably bound to this chip surface in the presence of 0.0005% tween-80 and the surface could be regenerated by standard methods (**SI Fig. S4D**). The characteristic binding profile of polymyxin B was also observed using OMVs non-specifically immobilized on the surface of a lipophilic LP chip (in the absence of tween-80), ruling out any potential effects of immobilization via amine-coupled polymyxin B or effects of

tween-80 (**SI Fig. S4F**). Note that higher concentrations were used to resolve the interaction above non-specific chip binding and the loss of polymyxin B to binding of plastics as discussed prior.

OMVs isolated from polymyxin-resistant bacteria (resistant-OMVs) were composed of modified lipid A (**SI Table S1** and **SI Fig. S6**) and were immobilized via amine-coupled polymyxin B to the surface of a C1 chip (**SI Fig. S4G**). Given the results of our studies here, the binding of resistant-OMVs to the amine-coupled polymyxin B surface (stably, at least within the time frames used here) is not surprising. OMVs have several advantages over whole cells and purified LPS. They can be produced in large batches and frozen for multiple experiments. OMVs can be isolated from potentially any genetic background (such as the two polymyxin-resistant strains used here), from potentially any gram-negative bacterial strains, and allow interrogation of types of LPS which are not commercially available (ex. modified lipid A species).

LP chip

LP chips (2D carboxymethyldextran surface, partially alkyl derivatized, Xantec Bioanalytics, Duesseldorf, Germany) were cleaned initially with two-20 sec pulses of 40 mM CHAPS (3-((3-cholamidopropyl) dimethylammonio)-1-propanesulfonate) with 10 sec dissociation at 30 µl/ml flow rate as recommended by the manufacturer. All LP-chip experiments were performed in 0.2 µm filtered 1x Dulbecco's PBS without $CaCl_2$ or $MgCl_2$ (Fisher Scientific), pH 7.4. Tween-80 was not compatible with this system, increasing the loss of polymyxin B due to non-specific binding[18]. Analysis and compartment temperature were set to 25°C. Mammalian vesicles from Expi293 cells were isolated as described[19] by harvesting supernatants and concentrating vesicles via ultra-centrifugation (120,000 x*g*, 4°C, 1h), washing, suspending pellet in PBS, quantifying by Bradford protein assay, and diluting to ~00.1 mg/mL in PBS. OMVs diluted in OMV buffer (described above) or mammalian vesicles were captured onto the chip for 60 sec (5 µL/ml flow rate) followed by a 300 sec stabilization period. Single cycle kinetics were performed as described for the C1 chip. To regenerate the chip, 40 mM CHAPS was injected to all channels for 180 sec (40 µL/ml), washed, 50 mM NaOH for 60 sec (40 µl/ml), buffer washed again, and finally four carry-over control steps. Regeneration of the LP chip was poor and build-up of material necessitated disposal of the chip.

SI Table S1. Proportion of modified LPS measured by LC-MS in OMV preparations used to quantify interactions with polymyxin B, PMBN, and brevicidine by SPR (full spectra are in Source Data file).

	**Lipid A species (%)**			
**OMVs**	**Unmodified**	**Single-modified**	**Double-modified**	**Total Modified**
Wild-type	100	0	0	0
pmrA^{G53E} batch 1	11.8	30.0	58.1	88.2
pmrA^{G53E} batch 2	8.7	21.2	70.1	91.3
+p*mcr1*	31.3	68.7	0	68.7

SI Table S2. Proportion of modified species identified from lipid A in SI Table S1 (full spectra are in Source Data file).

	Lipid A species (%)				
**OMVs**	**single pEtN**[1]	**single L-Ara4N**[2]	**double pEtN**	**double L-Ara4N**	**double L-Ara4N+pEtN**
pmrA^{G53E} batch 1	88.7	11.3	87.2	0.5	12.3
pmrA^{G53E} batch 2	93.5	6.5	80.3	0	19.7
+p*mcr1*	100	0	0	0	0

[1] pEtN – phosphoethanolamine lipid A modification
[2] L-Ara4N – 4-amino-4-deoxy-L-arabinose lipid A modification

SI Table S3. Half-life ($t_{1/2}$) and k_{off} of polymyxin B and brevicidine binding to immobilized wt-OMVs and resistant-OMVs measured by SPR at 25°C and 37°C with standard deviations.

Antibacterial	OMVs	Temp (°C)	$t_{1/2}$ (h)[1]	k_{off} (s^{-1})[2]
Polymyxin B	wt	25	6.86 (2.11)	3.25×10^{-5} (0.66×10^{-5})
	wt	37	7.27 (2.15)	2.84×10^{-5} (0.75×10^{-5})
Brevicidine	wt	25	1.09 (0.20)	1.8×10^{-4} (0.33×10^{-4})
	resistant[3]	25	1.17 (0.57)	1.96×10^{-4} (0.92×10^{-4})

[1] $t_{1/2}$ determined by chaser analysis from n≥3 independent replicates (see Source Data) as described in text

[2] k_{off} calculated from the measured residence time ($t_{1/2}$) from n≥3 independent replicates (see Source Data) as described[13]

[3] polymyxin B-resistant OMVs isolated from PmrA^{G53E} strain and strain carrying p*mcr*1 plasmid (n=2 independent replicates for each resistant mutant)

SI Table S4. Equilibrium binding constants (apparent-K_Ds) of polymyxin B and PMBN binding to immobilized wt-OMVs measured by SPR with standard deviations (n=4 for all except PMBN with resistant OMVs was n=3 independent replicates, see Source Data).

**Compound**	**OMVs**	**Apparent K_D [nM] (SD)**
Polymyxin B	WT (isolated reversible binding)	517 (449)
PMBN	WT	288 (153)
Polymyxin B	Resistant	273 (221)
PMBN	Resistant	371 (243)

SI Table S5. Strains and plasmid used in this study.

**Strain**	**Resistance gene**	**Source**	**GNEID**
E. coli BW25113	n/a	[20]	115
E. coli BW25113 *pmrA*^{G53E}	*n/a*	In-house	5164
E. coli BW25113 Δ*tolQ*	Kan-R	[20]	6119
E. coli BW25113 Δ*tolQ pmrA*^{G53E}	Kan-R	In-house	6120
S. aureus USA300	n/a	ATCC (BAA-1556)	23

**Plasmid**	**Description**	**Resistance gene**	**Source**
pBAD24-*mcr*1	Constitutive expression of *mcr1*	Carb-R	in house

SI References

1. Jung, L. S., Campbell, C. T., Chinowsky, T. M., Mar, M. N. & Yee, S. S. Quantitative Interpretation of the Response of Surface Plasmon Resonance Sensors to Adsorbed Films. *Langmuir* **14**, 5636–5648 (1998).

2. Karlsson, R., Roos, H., Fägerstam, L. & Persson, B. Kinetic and Concentration Analysis Using BIA Technology. *Methods* **6**, 99–110 (1994).

3. Karlsson, R. Biosensor binding data and its applicability to the determination of active concentration. *Biophys. Rev.* **8**, 347–358 (2016).

4. Theretz, A., Ranck, J.-L. & Tocanne, J.-F. Polymyxin B-induced phase separation and acyl chain interdigitation in phosphatidylcholine/phosphatidylglycerol mixtures. *Biochim. Biophys. Acta (BBA) - Biomembr.* **732**, 499–508 (1983).

5. Wofsy, C. & Goldstein, B. Effective Rate Models for Receptors Distributed in a Layer above a Surface: Application to Cells and Biacore. *Biophys. J.* **82**, 1743–1755 (2002).

6. Goldstein, B., Coombs, D., He, X., Pineda, A. R. & Wofsy, C. The influence of transport on the kinetics of binding to surface receptors: application to cells and BIAcore. *J. Mol. Recognit.* **12**, 293–299 (1999).

7. Sjoelander, S. & Urbaniczky, C. Integrated fluid handling system for biomolecular interaction analysis. *Anal. Chem.* **63**, 2338–2345 (1991).

8. Goldstein, B. & Dembo, M. Approximating the effects of diffusion on reversible reactions at the cell surface: ligand-receptor kinetics. Biophys. J. **68**, 1222–1230 (1995).

9. Johnson, K. A., Simpson, Z. B. & Blom, T. Global Kinetic Explorer: A new computer program for dynamic simulation and fitting of kinetic data. *Anal. Biochem.* **387**, 20–29 (2009).

10. Paracini, N., Clifton, L. A., Skoda, M. W. A. & Lakey, J. H. Liquid crystalline bacterial outer membranes are critical for antibiotic susceptibility. *Proceedings of the National Academy of Sciences* **1069**, 201803975–8 (2018).

11. Dejeu, J. *et al.* Impact of Conformational Transitions on SPR Signals - Theoretical Treatment and Application in Small Analytes/Aptamer Recognition. *J. Phys. Chem. C* **122**, 21521–21530 (2018).

12. Velkov, T., Thompson, P. E., Nation, R. L. & Li, J. Structure−Activity Relationships of Polymyxin Antibiotics. *Journal of Medicinal Chemistry* **53**, 1898–1916 (2010).

13. Quinn, J. G., Pitts, K. E., Steffek, M. & Mulvihill, M. M. Determination of Affinity and Residence Time of Potent Drug-Target Complexes by Label-free Biosensing. Journal of

Medicinal Chemistry **61**, 5154–5161 (2018).

14. Mastronarde, D. N. SerialEM: A Program for Automated Tilt Series Acquisition on Tecnai Microscopes Using Prediction of Specimen Position. *Microsc Microanal* **9**, 1182–1183 (2003).

15. Punjani, A., Rubinstein, J. L., Fleet, D. J. & Brubaker, M. A. cryoSPARC: algorithms for rapid unsupervised cryo-EM structure determination. *Nat Methods* **14**, 290–296 (2017).

16. Hankins, J. V., Madsen, J. A., Needham, B. D., Brodbelt, J. S. & Trent, M. S. The Outer Membrane of Gram-Negative Bacteria: Lipid A Isolation and Characterization. in vol. 966 239–258 (Humana Press, 2012).

17. Anderluh, G., Beseničar, M., Kladnik, A., Lakey, J. H. & Maček, P. Properties of nonfused liposomes immobilized on an L1 Biacore chip and their permeabilization by a eukaryotic pore-forming toxin. *Anal Biochem* **344**, 43–52 (2005).

18. Karvanen, M., Malmberg, C., Lagerbäck, P., Friberg, L. E. & Cars, O. Colistin Is Extensively Lost during Standard In Vitro Experimental Conditions. *Antimicrobial Agents and Chemotherapy* **61**, 131–9 (2017).

19. Théry, C., Amigorena, S., Raposo, G. & Clayton, A. Isolation and Characterization of Exosomes from Cell Culture Supernatants and Biological Fluids. *Curr Protoc Cell Biology* 3.22.1-3.22.29 (2006) doi:10.1002/0471143030.cb0322s30.

20. Wang, X. et al. Development of an SPR-based binding assay for characterization of anti-CD20 antibodies to CD20 expressed on extracellular vesicles. Anal Biochem **646**, 114635 (2022).

21. Baba, T. et al. Construction of Escherichia coli K-12 in-frame, single-gene knockout mutants: the Keio collection. *Molecular Systems Biology* **2**, 473–11 (2006).
