# Supplementary material for: Potent activity of polymyxin B is associated with long-lived super-stoichiometric accumulation mediated by weak-affinity binding to lipid A
Source: Nat Commun. 2024 Jun 3;15:4733. doi: 10.1038/s41467-024-49200-5 (PMC11148078; doi:10.1038/s41467-024-49200-5)
Supplement: Supplementary file 4 — Source Data [file 41467_2024_49200_MOESM4_ESM.zip › Source data file fig1C.pdf]

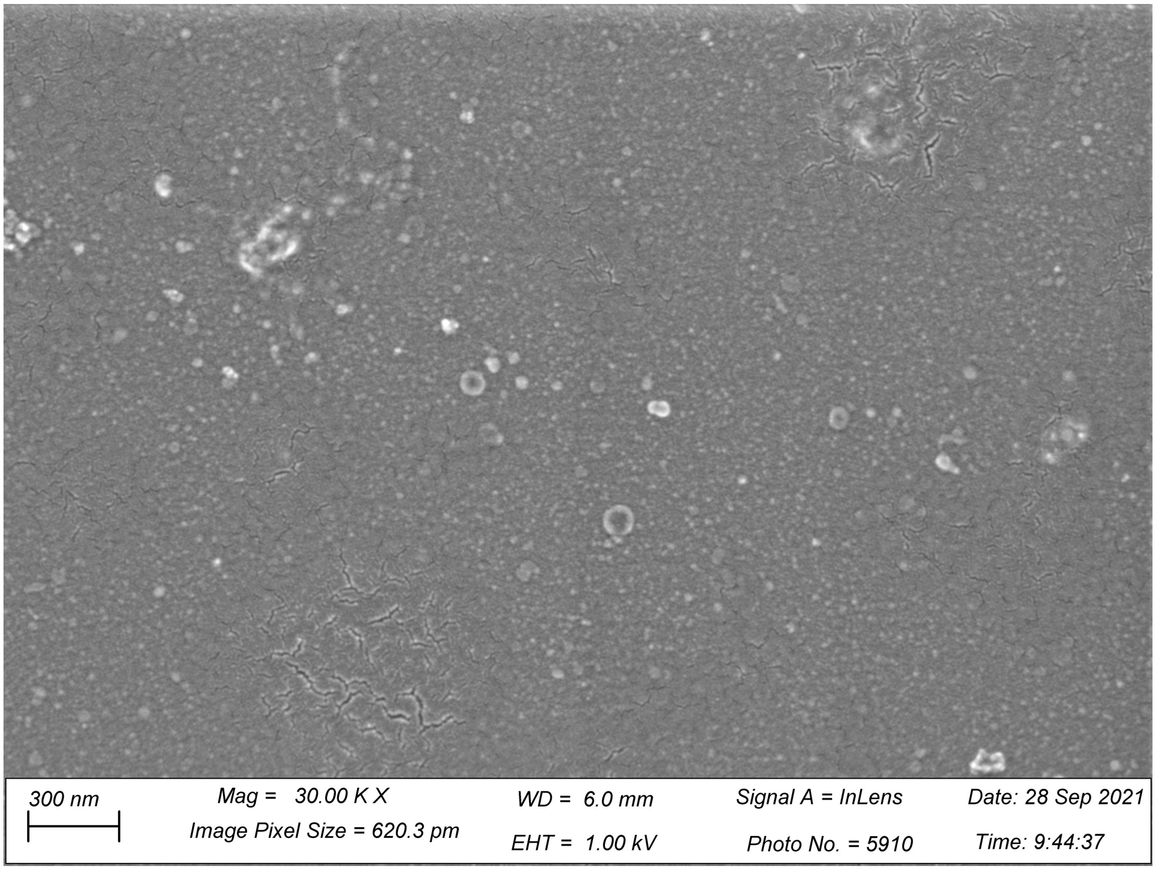
300 nm
Mag = 30.00 K X
Image Pixel Size = 620.3 pm
WD = 6.0 mm
EHT = 1.00 kV
Signal A = InLens
Photo No. = 5910
Date: 28 Sep 2021
Time: 9:44:37
