# Supplementary material for: Potent activity of polymyxin B is associated with long-lived super-stoichiometric accumulation mediated by weak-affinity binding to lipid A
Source: Nat Commun. 2024 Jun 3;15:4733. doi: 10.1038/s41467-024-49200-5 (PMC11148078; doi:10.1038/s41467-024-49200-5)
Supplement: Supplementary file 4 — Source Data [file 41467_2024_49200_MOESM4_ESM.zip › Spectra (SI Fig S6, SI Table S1, and SI Table S2)/Source Data File Descriptions (SI Fig S6, SI Table S1, and SI Table S2).pdf]

Data Key for Source Data files for SI Fig. S6, SI Table S1, and SI Table S2

**File name**	**Sample**
Dec01_HKLA_07.raw	Lipid A WT1
Dec01_KHLA_09.raw	*pmrA* Batch 1
18Feb22_Kerry_Kasia_05.raw	*pmrA* Batch 2
18Feb22_Kerry_Kasia_03.raw	+p*mcr1*
